# Supplementary material for: Aflatoxin B1 Increases Soluble Epoxide Hydrolase in the Brain and Induces Neuroinflammation and Dopaminergic Neurotoxicity
Source: Int J Mol Sci. 2023 Jun 9;24(12):9938. doi: 10.3390/ijms24129938 (PMC10298596; doi:10.3390/ijms24129938)
Supplement: Supplementary file 1 [file ijms-24-09938-s001.zip › ijms-2444236-supplementary.pdf]

Supplementary Materials

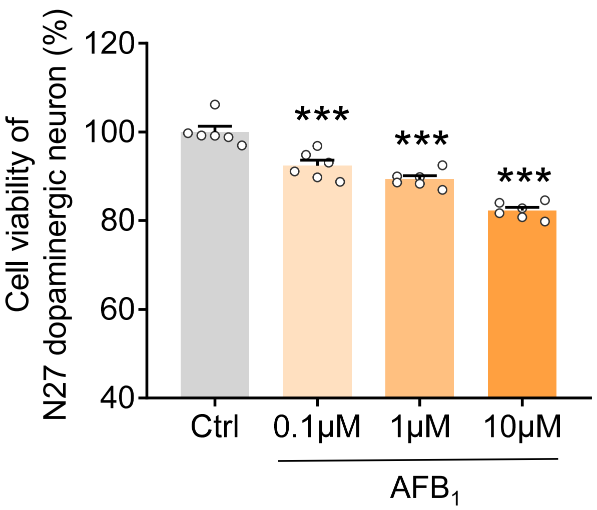

Figure S1. Cell viability of N27 rat dopaminergic neuron under AFB_1 treatment. The N27 rat dopaminergic neuron was treated with AFB_1 (0.1-10 μM) for 24 hours. The results are expressed as mean ± SEM. n = 6 wells per group. Statistical significance was determined using one-way ANOVA or Kruskal-Wallis test on Ranks. *** *P* < 0.001 vs Ctrl.

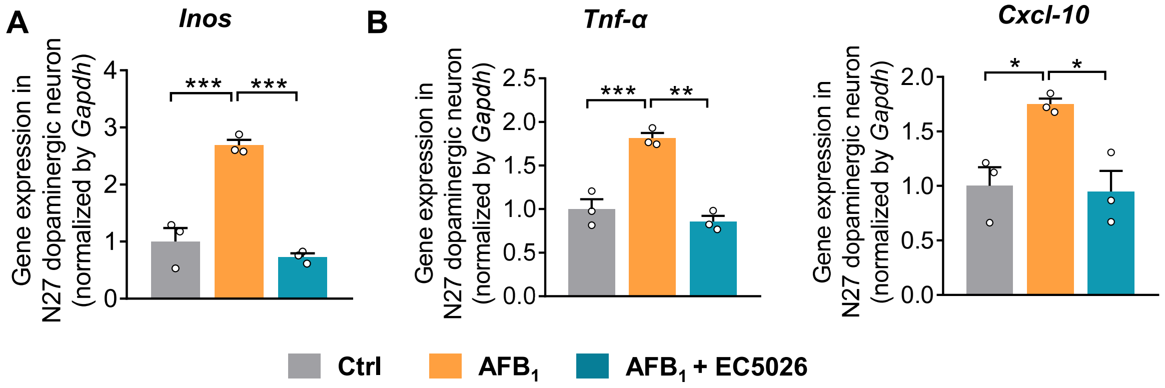

Figure S2. sEH inhibitor EC5026 blocks the AFB_1-upregulated oxidative and pro-inflammatory markers in N27 rat dopaminergic neurons. The N27 rat dopaminergic neuron was treated with AFB_1 (10 μM) with or without EC5026 (0.5 μM) for 24 hours. Gene expression of (**A**) oxidative marker *Inos* and (**B**) pro-inflammatory cytokines *Tnf-α* and *Cxcl10* in N27 rat dopaminergic neuron. The results are expressed as mean ± SEM. n = 3 wells per group. Statistical significance was determined using one-way ANOVA or Kruskal-Wallis test on Ranks. * *P* < 0.05, ** *P* < 0.01, *** *P* < 0.001.

Table S1. Sequences of primers in quantitative PCR

Gene	Forward primer	Reverse primer
**Mouse primer information**		
Gapdh	AGGTCGGTGTGAACGGATTTG (Tm = 62.6°C)	TGTAGACCATGTAGTTGAGGTCA (Tm = 62.6°C)
Iba-1	ATCAACAAGCAATTCCTCGATGA (Tm = 60.3°C)	CAGCATTCGCTTCAAGGACATA (Tm = 60.7°C)
Il-1β	GCAACTGTTCCTGAACTCAACT (Tm = 60.7°C)	ATCTTTTGGGGTCCGTCAACT (Tm = 61.4°C)
Mcp-1	TTAAAAACCTGGATCGGAACCAA (Tm = 60.1°C)	GCATTAGCTTCAGATTTACGGGT (Tm = 60.7°C)
Csf-2	GGCCTTGGAAGCATGTAGAGG (Tm = 62.5°C)	GGAGAACTCGTTAGAGACGACTT (Tm = 61.1°C)
Cxcl-10	CCAAGTGCTGCCGTCATTTTC (Tm = 62.4°C)	GGCTCGCAGGGATGATTTCAA (Tm = 62.8°C)
Snca	GGGAGTCCTCTATGTAGGTTCC (Tm = 60.4°C)	TCCAACATTTGTCACTTGCTCT (Tm = 60.1°C)
Tyrosine hydroxylase	GTCTCAGAGCAGGATACCAAGC (Tm = 62.1°C)	CTCTCCTCGAATACCACAGCC (Tm = 61.7°C)
Ephx2	GCGTTCGACCTTGACGGAG (Tm = 63.0°C)	TGTAGCTTTCATCCATGAGTGGT (Tm = 61.3°C)
**Rat primer information**		
Gapdh	GCCATCAACGACCCCTTCAT (Tm = 60.4°C)	CGCCTGCTTCACCACCTTCT (Tm = 62.7°C)
Inos	CCTTGTTCAGCTACGCCTTC (Tm = 58.9°C)	GGTATGCCCGAGTTCTTTCA (Tm = 57.6°C)
Tnf-α	CCCTCACACTCAGATCATCTTCT (Tm = 59.2°C)	GCTACGACGTGGGCTACAG (Tm = 60.2°C)
Cxcl-10	TGCAAGTCTATCCTGTCCGC (Tm = 59.8°C)	TCTTTGGCTCACCGCTTTCA (Tm = 60.1°C)
